# Injury Incidence in Community-Based Walking Football: A Four-Month Cohort Study of 6000+ Hours of Play

**DOI:** 10.3390/sports13050150

**Published:** 2025-05-19

**Authors:** Alfie G. Price, Bradley Sprouse, Avinash Chandran, John Hough, Philip J. Hennis, Ali Ahmed, Ian Varley

**Affiliations:** 1 School of Science and Technology, Nottingham Trent University, Nottingham NG11 8NS, UK; alfie.price@ntu.ac.uk (A.G.P.); bradley.sprouse@ntu.ac.uk (B.S.); john.hough@ntu.ac.uk (J.H.); philip.hennis@ntu.ac.uk (P.J.H.); 2 Datalys Center for Sports Injury Research and Prevention, Indianapolis, IN 46220, USA; avinashc@datalyscenter.org; 3 University Hospitals of Derby and Burton NHS Foundation Trust, Derby DE22 3NE, UK; ali.ahmed4@nhs.net

**Keywords:** walking football, walking soccer, ageing, sports injury, exercise, health, physical activity, recreational sports, older adults, healthy ageing

## Abstract

Walking Football is a growing adapted sport offering a viable alternative to traditional exercise for middle-aged and older adults. While rule modifications aim to reduce injury risk, this has yet to be established. This study conducted injury surveillance in community-based Walking Football to determine injury incidence and characteristics in training and matches. A four-month observational cohort study remotely tracked injuries and exposure time across seven Walking Football clubs in England using a sub-elite injury surveillance framework. Injuries were classified as medical attention (requiring on-field attention without subsequent absence) or time-loss (≥1 day of participation absence). Injury incidence was calculated per 1000 h of play. Across 6364.55 h of exposure, 45 injuries were reported: 30 (66.7%) medical attention and 15 (33.3%) time-loss injuries. Injury incidence was 5.3 [1.5–11.5] per 1000 h in training (medical attention: 3.3 [0.8–7.3]; time-loss: 2.0 [0.5–4.5]) and 37.6 [8.7–83.9] per 1000 h in matches (medical attention: 28.9 [5.8–66.6]; time-loss: 8.7 [0–23.2]). Match injury incidence was significantly higher than training (rate ratio: 7.1 [1.3–31.4]). Findings suggest that injury incidence in community-based Walking Football is low, supporting its safety and potential as a sustainable physical activity strategy for middle-aged and older adults.

## 1. Introduction

Walking Football is a modified version of traditional Association Football designed to accommodate middle-aged and older adults by reducing physical demands while preserving the core components of the game. Rule adaptations, such as prohibiting running and limiting physical contact, aim to make the sport accessible to individuals with physical limitations or pre-existing health conditions [1]. As such, Walking Football has the potential to address challenges linked to the global age-related decline in physical activity [2,3]. Despite widespread assumptions that these rule modifications lower the risk of injury [4], and given the sport’s dynamic nature [5], the actual injury incidence remains unclear.

Although some studies have reported injuries occurring during Walking Football interventions [6,7], there is limited evidence on the incidence and characteristics of injuries sustained by habitual participants. This gap in knowledge contrasts with Association Football, where injury epidemiology has been established at a professional [8], amateur [9], and recreational level [10]. Given the demographic differences and reduced intensity compared to Association Football, this renders direct comparisons between the two sports difficult. Conducting sport-specific injury surveillance in Walking Football is therefore crucial for understanding its safety profile, identifying injury trends, and informing strategies to enhance player welfare [11,12].

Walking Football is a rapidly growing sport [13], attracting an older population that is therefore more likely to include individuals with pre-existing health conditions, reinforcing the need to better understand the sport’s safety profile. Despite its popularity, Walking Football is commonly played in settings without dedicated medical support or standardised injury reporting systems. Consequently, there is a pressing need to establish baseline injury incidence and characteristics within this population. This will optimise player welfare by informing injury prevention strategies and guiding governance within the sport. Understanding injury incidence is also essential for healthcare professionals, community sports organisers, and potential participants in making informed, evidence-based decisions regarding participation. Currently, the lack of large-scale surveillance data limits meaningful discussions regarding injury risk and management in community-based Walking Football.

This study aims to address these gaps by investigating injury incidence in Walking Football at a community level. Additionally, it seeks to analyse the characteristics of injuries, including their types, causes, and anatomical locations. By establishing a clearer picture of the injury landscape in community-based Walking Football, this research will contribute to player safety efforts, support informed decision-making, and provide a foundation for evaluating future injury prevention strategies tailored to the needs of Walking Football participants.

## 2. Materials and Methods

### 2.1. Study Design

**Figure 1:**
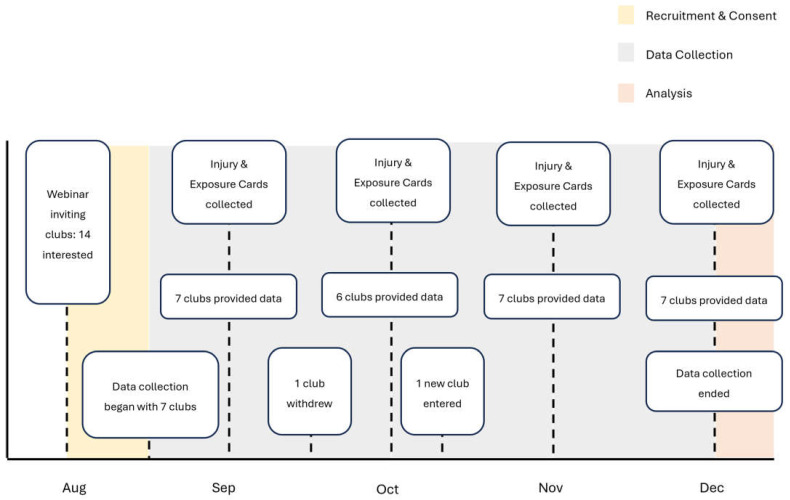
Timeline of the study: Recruitment and Consent, Data Collection, and Analysis phases.

This study employed a longitudinal, observational cohort design to monitor self-reported injuries during training and matches in Walking Football clubs across England. A nationwide webinar was firstly conducted in collaboration with The Football Association of England (The FA) to introduce the study to Walking Football clubs across the country. The FA is the governing body for Association Football in England, who invited all registered Walking Football clubs to attend, representing a convenience sampling approach. The webinar provided an overview of the research objectives, data collection requirements, and participation expectations. The inclusion process operated at two levels. First, clubs were eligible to participate if they were based in England and delivered organised Walking Football sessions. Participation was open to all clubs expressing interest following the nationwide webinar. Second, within participating clubs, all individuals attending sessions were eligible for inclusion provided they gave informed consent for injury data collection. Fourteen clubs initially expressed interest, with seven clubs ultimately providing data throughout the study period (August–November 2024). While one club withdrew from the study partway through the observation period, a replacement club, which had also heard about the study via The FA, was recruited and joined shortly afterwards, following the same data collection procedures as the initial clubs (Figure 1). Data from the club that withdrew were not included in the final analysis. Participating clubs were geographically distributed across the North West (*n* = 2), East (*n* = 1), South East (*n* = 2), and South West (*n* = 2) regions. Each club operated open sessions whereby players could attend based on availability. The sample size was relative and convenient in nature, determined by the number of players attending participating clubs’ sessions and the number who provided informed consent for injury data collection.

Ethical approval for the study was granted by the Nottingham Trent University Non-Invasive Ethical Review Committee (application ID: 1895303). All participants provided informed consent before injury data collection commenced. To maintain confidentiality, no personal identifiers were reported, and injury data were collected exclusively from consenting players.

### 2.2. Data Collection

Data were collected in accordance with international consensus recommendations for epidemiological research in professional football [14], with adaptations made to align with a sub-elite injury surveillance framework [15]. Time-loss injuries were defined as injuries resulting in a removal from play and an estimated absence from future participation for at least one day, while medical attention injuries were defined as injuries that required on-field attention and a pause in play but did not lead to subsequent absence [16]. Furthermore, matches were defined as fixtures against external teams (e.g., league games), while training encompassed all other organised Walking Football activities. The clubs included teams across multiple age categories (40 years+ to 70 years+) and formats (men’s, women’s, and mixed teams). Given the diverse nature of Walking Football leagues, match rules may have varied across clubs and deviated slightly from The FA’s standardised regulations for the sport [17].

Data were collected either at an individual level or on a club-wide basis. Two clubs adopted an individual reporting approach, whereby consenting players recorded their own injuries and exposure. In the remaining five clubs, a designated individual, such as a coach or team captain, was responsible for recording injuries and tracking exposure for all players. These designated individuals completed monthly exposure reports to document playing time across training and match activities. In clubs where a designated individual managed remote data collection, consent was reaffirmed at the time of each recorded injury. A standardised injury surveillance form documented injury occurrences and characteristics such as causes, types, locations, activities at the time of injury, re-injury rates, associations with pre-existing conditions, and playing surface (Supplementary Materials File S1: Injury Surveillance Form). This form was developed using international consensus guidelines for epidemiological studies [14] and adapted based on a sub-elite injury surveillance framework [15]. Additionally, a standardised exposure tracking form was used to record playing time (Supplementary Materials File S2: Exposure Tracking Form). Blank injury and exposure forms were distributed electronically to participating clubs at the beginning of the study, with clubs completing and returning one exposure form per month and one injury form per reported case. All returned forms were reviewed for completeness. No forms were excluded from analysis, although clarification was occasionally sought from club representatives to ensure accuracy and completeness of data.

### 2.3. Analysis

Injury incidence was calculated as the number of injuries per 1000 h of exposure. Incidence rates were reported with 95% confidence intervals, derived using a negative binomial distribution to account for overdispersion, and employing a parametric bootstrap from 10,000 simulated counts, sampling at an aggregated level (Supplementary Materials File S3: Injury Incidence Calculation Code). An injury rate ratio (IRR) with accompanying 95% confidence intervals was also calculated, employing a similar parametric bootstrapped negative binomial approach, to estimate the relative difference between training and matches (Supplementary Materials File S4: Injury Rate Ratio Calculation Code). The IRR was deemed statistically significant if its 95% confidence intervals excluded 1.00 [18]. Injury incidence values and IRR are rounded to one decimal place for ease of interpretation. All analyses were conducted in RStudio (version 2024.09.0-375).

When reporting injury characteristics, injuries that could lead to indirect identification were combined under the category ‘Other’. An asterisk was placed at the bottom of the table to indicate the specific categories included in ‘Other’, along with categories with zero reported cases, ensuring that the exact number of injuries in categories with one or more reported cases remained undisclosed to maintain participant anonymity. The category ‘Unique Injuries’ referred to injuries that did not fall under the pre-defined categories set out in the injury surveillance form (Supplementary Materials File S1: Injury Surveillance Form).

## 3. Results

### 3.1. Overall Injury Incidence

The total recorded exposure was 6364.55 h of Walking Football play over 5589 instances of attendance for the seven participating clubs (mean: 798; median: 226; range: 39–2957 instances of attendance). In the five clubs who adopted a club-wide approach to data collection, there were a total of 389 Walking Football sessions (mean: 78; median: 39; range: 23–205 sessions) consisting of 220 training sessions (mean: 44; median: 15; range: 8–133 training sessions) and 169 matches (mean: 34; median: 27; range: 15–72 matches). There was a total of 45 injuries documented throughout the study period. Of these, 30 (66.7%) were categorised as medical attention injuries, while the remaining 15 (33.3%) resulted in time-loss. The overall injury incidence was 7.1 [2.0–14.8] injuries per 1000 h of exposure. Medical attention injuries occurred at an incidence of 4.7 [1.3–10.2] per 1000 h, whereas time-loss injuries were recorded at 2.4 [0.5–5.2] per 1000 h.

### 3.2. Injury Incidence in Training

Total training exposure amounted to 6019 h. Over this period, 32 injuries were reported, with 20 (62.5%) classified as medical attention injuries and 12 (37.5%) as time-loss injuries. The overall training injury incidence was 5.3 [1.5–11.5] injuries per 1000 h, with medical attention injuries occurring at 3.3 [0.8–7.3] per 1000 h and time-loss injuries at 2.0 [0.5–4.5] per 1000 h.

### 3.3. Injury Incidence in Matches

**Figure 2:**
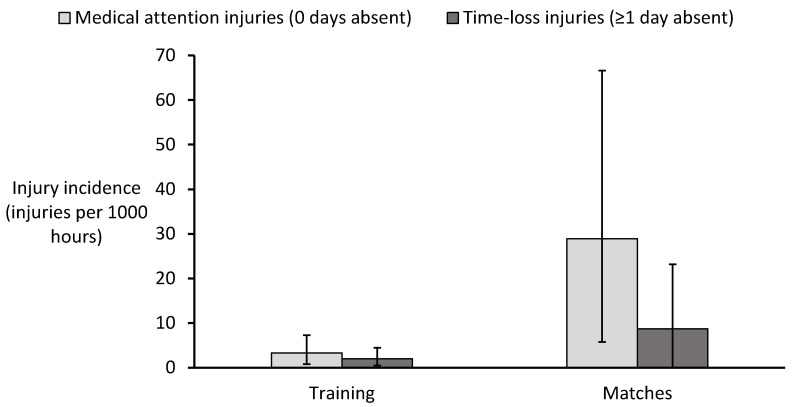
Incidence of medical attention and time-loss injuries for training and matches in Walking Football. Error bars represent 95% confidence intervals.

A total of 345.55 h of match play were recorded, during which 13 injuries occurred. Of these, 10 (76.9%) required medical attention, while 3 (23.1%) resulted in time-loss. The overall match injury incidence was calculated at 37.6 [8.7–83.9] injuries per 1000 h, with medical attention injuries occurring at an incidence of 28.9 [5.8–66.6] per 1000 h and time-loss injuries at 8.7 [0–23.2] per 1000 h (Figure 2). Match injury incidence was significantly greater in comparison to training when considering all injuries (IRR: 7.1 [1.3–31.4]) as well as medical attention (IRR: 8.7 [1.5–41.8]) and time-loss injuries (IRR: 4.4 [0–26.1]).

### 3.4. Injury Causes

Non-contact mechanisms accounted for most injuries as 60% of all recorded cases (Table 1). This trend was consistent across training (62.5%) and matches (54.5%). Contact-related injuries were observed at similar proportions in both settings, representing 25% of training injuries and 27.3% of match injuries.

### 3.5. Activity at Time of Injury

The most frequent activity associated with injury, both overall and within training and match settings, fell under the ‘Unique Injuries’ category (Table 2). This included incidents such as falling, sudden twisting movements, overstretching, and the foot becoming stuck in the turf. Kicking was responsible for 18.2% of all injuries, while running accounted for 11.4%.

### 3.6. Injury Types

Muscle strain/tear/rupture/cramps were the most prevalent injury type overall (26.7%) and were most frequent in match play (46.2%; Table 3). In training, the most reported injury type fell under the ‘Unique Injuries’ category (25%), which included superficial injuries such as grazes and shortness of breath.

### 3.7. Injury Location

The knee was the most frequently injured anatomical site, accounting for 37.2% of all injuries (Table 4). This pattern was consistent across training (40%) and match play (30.8%).

### 3.8. Pre-Existing Conditions, Re-Injuries, and Playing Surface

Of the 42 injuries for which this information was available, 4 (9.5%) were linked to pre-existing conditions. Re-injuries accounted for 6 of 36 injuries (16.7%). Within training, 3 of 29 injuries (10.3%) were associated with pre-existing conditions, while 6 of 26 injuries (23.1%) were classified as re-injuries. In match play, only 1 of 13 injuries (7.7%) was linked to a pre-existing condition, and no re-injuries were recorded. Regarding playing surfaces, none of the 45 reported injuries occurred on natural grass. The majority (88.9%) took place on artificial turf, while the remaining 5 cases were categorised under ‘Other’ surfaces.

## 4. Discussion

This study aimed to measure injury incidence and characteristics in community-based Walking Football to inform injury prevention strategies and optimise player welfare. The findings indicate an overall injury incidence of 7.1 [2.0–14.8] injuries per 1000 h of exposure, with medical attention and time-loss injury incidences of 4.7 [1.3–10.2] and 2.4 [0.5–5.2] per 1000 h, respectively. Most injuries (60%) were non-contact, with 18.2% occurring during kicking actions. Muscle strain/tear/rupture/cramps (26.7%) were the most common type of injury, while the knee (37.2%) was the most frequently affected location.

The primary finding was that the overall injury incidence in Walking Football was relatively low. This is encouraging given that age is a key risk factor for injury in football [19], and the study consisted of Walking Football participants aged 40 years+ up to 70 years+. Indeed, only one-third of total injuries resulted in time-loss, suggesting that most injuries did not hinder participation. All injuries could deter casual players at the community level, but the incidence of medical attention injuries remained low, indicating that the sport is safe for middle-aged and older adults.

## 5. Comparison with Recreational Football

Walking Football demonstrated a lower overall injury incidence (7.1 [2.0–14.8] injuries per 1000 h) compared to recreational Association Football in studies with an average age of participants of 30 years+ (12.4–71.7 injuries per 1000 h) [19,20,21,22], highlighting the role of the modified rules in reducing injury risk. The proportion of contact injuries was lower in Walking Football (25.7%) compared to Association Football leagues for players aged 30 years+ (81%) and 40 years+ (61%) in Switzerland [21], suggesting that Walking Football’s minimal contact rule may contribute to this reduced risk. The higher number of non-contact injuries reported could suggest that age may have an influence on injury mechanisms, with older players potentially more susceptible due to reduced physical resilience. However, further sport-specific research is required to substantiate this suggestion.

Injury incidence in Walking Football was significantly greater in matches (37.6 [8.7–83.9] injuries per 1000 h) than in training (5.3 [1.5–11.5] injuries per 1000 h) (IRR: 7.1 [1.3–31.4]), which was also a trend observed in recreational Association Football [19]. This may be due to the reduced intensity and competitiveness in training. However, training session content was not recorded, which could have influenced injury risk. Interestingly, in contrast to Association Football [19], Walking Football displayed a similar proportion of contact-related injuries in training and matches, reinforcing that its modified rules may be effective in limiting physicality and enhancing player safety.

Time-loss injury incidence in Walking Football was also lower than in recreational Association Football [19,20]. Specifically, time-loss injuries per 1000 h were lower in both training (2.0 [0.5–4.5] vs. 4.5 [4.2–8.7]) and matches (8.7 [0–23.2] vs. 24.7 [18.3–31.1]) compared to veteran Association Football players aged 32–69 years [19]. This suggests that Walking Football offers a safer alternative for middle-aged and older adults, with reduced speed and fewer rapid directional changes likely contributing to the lower injury incidence. Additionally, only 16.7% of injuries were re-injuries, significantly lower than the 32% reported in recreational Association Football [19], and only 9.5% were linked to pre-existing conditions. This finding is particularly important, as previous injuries and existing conditions often act as a barrier to sport participation in middle-aged and older adults [23,24]. The low percentage of injuries related to existing conditions suggests that Walking Football may have minimal impact on exacerbating such conditions, making it a potentially safer and more accessible option for exercise referral schemes and social prescriptions. This evidence supports the view that Walking Football may provide older adults with a viable means of engaging in physical activity without significant risk of injury related to their health status.

The most common injury location reported in a Turkish football tournament among public employees (average age 35 years) was the thigh (29%) [20], differing from the knee (30.8%) for match injuries in Walking Football. When considering both training and matches, the knee remained the most common site in Walking Football (37.2%), followed jointly by the ankle and lower leg (11.6% each). This suggests that injuries of the lower extremities are inherent to football participation. Indeed, lower limb injuries remain a common feature across all recreational football formats [10]. Furthermore, the most common injury type in the Turkish football tournament was muscle strain (40%) [20], similar to the present study for Walking Football matches (muscle strain/tear/rupture/cramps; 46.2%). The reason for this could be linked to movements inherent to the sport, such as kicking and directional changes [25], which are difficult to mitigate. Muscle strain/tear/rupture/cramps were more frequent in Walking Football matches compared to training, potentially due to faster movement speeds associated with increased competitive intensity. Conversely, kicking-related injuries were more frequent in training, perhaps due to greater ball exposure and more technical drills involving frequent passing and shooting.

## 6. Contextualising Injury Incidence Across Recreational Activities

The injury incidence of Walking Football appears relatively low when compared to other recreational activities. In a Finnish cohort of adults aged 15–74 years, self-reported annual trauma and overuse injury incidences per 1000 h were lower in activities such as swimming (1.0 [0.65–1.40]), walking (1.2 [1.0–1.3]), cycling (2.0 [1.7–2.5]), and gym training (3.1 [2.5–3.8]) compared to Walking Football (7.1 [2.0–14.8]), but comparable to racket sports like badminton (4.6 [3.1–6.8]), and tennis (4.7 [2.9–7.7]) [26]. Conversely, time-loss injury incidence in Walking Football (2.4 [0.5–5.2] injuries per 1000 h) was lower than in recreational running for middle-aged adults (average age 38–42 years; 7.6–33.0 injuries per 1000 h of exposure) [27,28,29,30]. These comparisons suggest that, although Walking Football carries some inherent risk, its modified format reduces the likelihood of injury, making it a relatively safe recreational sport. Moreover, the social aspects of team sports like Walking Football can help maintain motivation and foster long-term adherence, with participants often experiencing a stronger sense of enjoyment and social connection compared with traditional forms of exercise [31,32]. This positions Walking Football not only as a safe option, but also as a sustainable and engaging choice for middle-aged and older adults to increase their levels of physical activity.

## 7. Injury Prevention Considerations

Certain modifiable risk factors could be addressed to further reduce injuries. Tackles accounted for 15.6% of injuries in training, while running contributed to 16.7% of match injuries. Strengthening enforcement of the minimal contact rule in training and reinforcing the no-running rule in matches, particularly through clear guidance for coaches and referees, may help minimise these risks. Additionally, the most frequently reported activity at the time of injury for both training and matches were those under the ‘Unique Injuries’ category, including falls, overstretching, foot entrapment in turf, and sudden twisting movements, some of which are consistent with challenges in balance and proprioception associated with ageing [33]. Clubs could therefore consider implementing age-appropriate neuromuscular warm-up routines focused on mobility and lower-limb stability. However, while such routines have demonstrated injury prevention benefits in younger populations [34], there is limited research on their effectiveness and suitability for older adults. Adapted or simplified versions may offer similar protective benefits, but further study is warranted. In addition, for older participants with joint instability in the ankle or knee, using external joint supports may help reduce injury risk during play [35].

Given that most injuries occurred on artificial grass (88.9%), alongside reports of foot entrapment, it is important to consider the potential role of footwear in injury risk [36]. Participants in Walking Football, many of whom may be returning to sport after a long absence or playing for the first time, may be less aware of the necessity of using surface-appropriate footwear to ensure sufficient grip and avoid entrapment. Providing players with clear guidance on suitable footwear for different surfaces may therefore help to enhance player safety.

Finally, the greater concentration of injuries in players unaccustomed to football activity in the early training period of recreational Association Football [37] suggests that a gradual introduction to Walking Football may be important to minimise injury risk, particularly for those entering after a period of inactivity. While some degree of risk is inherent in any sport, the overall injury incidence suggests that these risks are acceptable given the potential physical and social benefits Walking Football provides [1,38]. Nevertheless, integrating evidence-based strategies at the community level could help enhance player safety and support long-term participation.

## 8. Strengths and Limitations

This study is the first to conduct injury surveillance in community-based Walking Football using standardised methodology, allowing for future comparisons and long-term monitoring of safety strategies. The dataset, collected from multiple clubs across England, provides a representative overview of injury characteristics. Additionally, real-time injury tracking minimises recall bias, enhancing data reliability. Future research would benefit from large-scale, multi-country injury surveillance monitoring in Walking Football to assess the impact of rule variations on injury risk.

Nonetheless, some limitations should be acknowledged. Firstly, injury data were self-reported, potentially introducing variability in reporting accuracy; however, remote injury surveillance was necessary due to resource constraints in grassroots sports. Future research could include comparative in-person studies to assess the reliability of remote self-reporting methods and to determine best practices for accurate injury surveillance. Secondly, we were unable to stratify injury incidence by age and gender, as clubs operated open sessions where player attendance varied based on availability, making it impossible to determine the total number of participants and their demographic distribution using our study design. Furthermore, conducting comprehensive participant characterisation would have placed a substantial administrative burden on club organisers and the designated individual selected for data collection, risking reduced participation in the study. We acknowledge that collecting such data would have strengthened the analysis by allowing for subgroup comparisons and more precise generalisability of findings. Future research would benefit from considering feasible and low-burden methods for capturing large-scale participant demographics in community-based sports settings. Additionally, training session content was not recorded, meaning variations in training structure across clubs could not be accounted for despite influencing injury risk. Documenting session content would have required an additional burden on coaches or monitoring by the research team across many community-based sites, which was not feasible within the scope and resources of this study. Understanding the nature and intensity of training sessions could help identify high-risk activities, and future studies should consider pragmatic methods to capture this information. Finally, while descriptive data on the playing surface for each injury was reported, we were unable to collect exposure information for each surface type. Therefore, it cannot be determined whether certain surfaces influenced injury incidence or whether surface-related injury patterns reflect differences in exposure. Future studies should incorporate surface-specific exposure tracking to draw conclusions about the relative safety of natural grass versus artificial turf for Walking Football.

## 9. Conclusions

This study delivers the first comprehensive, community-based injury surveillance data for Walking Football, filling a critical gap in the literature. The findings indicate that Walking Football presents a lower injury incidence than recreational Association Football in veteran players, reinforcing its potential as a safe, inclusive and health-promoting sport for middle-aged and older adults. These insights offer much needed reassurance to healthcare professionals, potential players and community organisers, and position Walking Football as a credible option for long-term health promotion. Furthermore, its suitability for social prescribing and exercise referral schemes underscores its growing public health relevance. Future research should continue to evaluate injury trends and characteristics, alongside assessing the effectiveness of rule modifications in ensuring player safety while maintaining the sport’s appeal.

## Figures and Tables

**Table 1:** Cause of injury in Walking Football (injury frequency and proportion [%]).

Cause	Overall (*n* = 35)	Training (*n* = 24)	Matches (*n* = 11)
Contact	9 (25.7%)	6 (25.0%)	3 (27.3%)
Non-Contact	21 (60.0%)	15 (62.5%)	6 (54.5%)
Cumulative	5 (14.3%)	3 (12.5%)	2 (18.2%)

**Table 2:** Activities associated with injuries (injury frequency and proportion [%]).

Activity	Overall (*n* = 44)	Training (*n* = 32)	Matches (*n* = 12)
Unique Injuries	13 (29.5%)	9 (28.1%)	4 (33.3%)
Kicking	8 (18.2%)	7 (21.9%)	1 (8.3%)
Tackled	6 (13.6%)	5 (15.6%)	1 (8.3%)
Cumulative	5 (11.4%)	3 (9.4%)	2 (16.7%)
Running	5 (11.4%)	3 (9.4%)	2 (16.7%)
Collision	4 (9.1%)	2 (6.3%)	2 (16.7%)
Other *	3 (6.8%)	3 (9.4%)	0 (0%)

* Direct blow, heading, jumping/landing, tackling.

**Table 3:** Distribution of injury types (injury frequency and proportion [%]).

Type	Overall (*n* = 45)	Training (*n* = 32)	Matches (*n* = 13)
Muscle Strain/Tear/Rupture/Cramps	12 (26.7%)	6 (18.8%)	6 (46.2%)
Unique Injuries	10 (22.2%)	8 (25.0%)	2 (15.4%)
Haematoma/Contusion/Bruise	6 (13.3%)	4 (12.5%)	2 (15.4%)
Pain (Undiagnosed)	6 (13.3%)	4 (12.5%)	2 (15.4%)
Cartilage/Disc/Meniscus	4 (8.9%)	4 (12.5%)	0 (0%)
Other *	7 (15.6%)	6 (18.8%)	1 (7.7%)

* Bone fracture, bursitis/impingement/synovitis, concussion, ligament sprain/tear/rupture, tendon injury/rupture/tendinopathy.

**Table 4:** Injury locations (injury frequency and proportion [%]).

Location	Overall (*n* = 43)	Training (*n* = 30)	Matches (*n* = 13)
Knee	16 (37.2%)	12 (40.0%)	4 (30.8%)
Ankle	5 (11.6%)	3 (10.0%)	2 (15.4%)
Lower Leg	5 (11.6%)	3 (10.0%)	2 (15.4%)
Head/Face	4 (9.3%)	4 (13.3%)	0 (0%)
Unique Injuries	0 (0%)	0 (0%)	0 (0%)
Other *	13 (30.2%)	8 (26.7%)	5 (38.5%)

* Abdomen, Achilles tendon, anterior thigh, elbow, foot/toe, forearm, hip/groin, low back, medial thigh, neck/cervical spine, pelvis/sacrum, posterior thigh, shoulder/clavicle, sternum/ribs/upper back, upper arm, wrist/hand/finger/thumb.

## Data Availability

The data that support the findings of this study are available from the corresponding author upon reasonable request.

## Supplementary Materials

The following supporting information can be downloaded at https://www.mdpi.com/article/10.3390/sports13050150/s1, Supplementary Materials File S1: Injury Surveillance Form—A standardised form used to document injury occurrences, including causes, types, locations, activities at the time of injury, re-injuries, associations with pre-existing conditions, and playing surface. Supplementary Materials File S2: Exposure Tracking Form—A standardised form used to record playing time during training and matches for injury incidence calculations. Supplementary Materials File S3: Injury Incidence Calculation Code—The RStudio code used to calculate injury incidence with 95% confidence intervals. Supplementary Materials File S4: Injury Rate Ratio Calculation Code—The RStudio code used to compute the injury rate ratio (IRR) and its 95% confidence intervals for comparing training and match injury incidence.
