# Supplementary material for: Injury Incidence in Community-Based Walking Football: A Four-Month Cohort Study of 6000+ Hours of Play
Source: Sports (Basel). 2025 May 19;13(5):150. doi: 10.3390/sports13050150 (PMC12115786; doi:10.3390/sports13050150)
Supplement: Supplementary file 1 [file sports-13-00150-s001.zip › Supplementary Materials File S1 - Injury Surveillance Form.pdf]

Walking Football Injury Surveillance Study (Injury Card)

Details

Team Name:

Gender: ☐ Male ☐ Female ☐ Non-Binary ☐ Prefer Not To Say

Age Group: ☐ 31-40 ☐ 41-50 ☐ 51-60 ☐ 61-70 ☐ 71+

Injury Details

Time loss injury? ☐ Yes ☐ No

Date of Injury:

Estimated Injury Severity (Days Lost):

Occasion

☐ Match-Play

☐ Training

☐ Other Club Related Activity

Cause of Injury

☐ Contact

☐ Non-Contact

☐ Cumulative

Type of Injury

☐ Muscle Strain/Tear/Rupture/Cramps	☐ Ligament Sprain/Tear/Rupture	☐ Tendon Injury/Rupture/Tendinopathy
☐ Haematoma/Contusion/Bruise	☐ Concussion	☐ Cartilage/Disc/Meniscus
☐ Bursitis/Impingement/Synovitis	☐ Bone Fracture	☐ Pain (Undiagnosed)
☐ Other (Please State)		

Location of Injury

☐ Abdomen	☐ Achilles Tendon	☐ Ankle
☐ Anterior Thigh	☐ Elbow	☐ Foot/Toe
☐ Forearm	☐ Head/Face	☐ Hip/Groin
☐ Knee	☐ Low Back	☐ Lower Leg
☐ Medial Thigh	☐ Neck/Cervical Spine	☐ Pelvis/Sacrum
☐ Posterior Thigh	☐ Shoulder/Clavicle	☐ Sternum/Ribs/Upper Back
☐ Upper Arm	☐ Wrist/Hand/Finger/Thumb	

Activity at Time of Injury

☐ Tackled	☐ Tackling	☐ Collision
☐ Running	☐ Jumping/Landing	☐ Kicking
☐ Heading	☐ Direct Blow	☐ Cumulative
☐ Other (Please State)		

Surface

☐ Grass ☐ Artificial Turf ☐ Other

Re-Injury

Is it a re-injury? ☐ Yes ☐ No

Existing Conditions

Is the injury linked to an existing condition (e.g. arthritis)? ☐ Yes ☐ No

If yes, please state the existing condition:
