# Supplementary material for: Injury Incidence in Community-Based Walking Football: A Four-Month Cohort Study of 6000+ Hours of Play
Source: Sports (Basel). 2025 May 19;13(5):150. doi: 10.3390/sports13050150 (PMC12115786; doi:10.3390/sports13050150)
Supplement: Supplementary file 1 [file sports-13-00150-s001.zip › Supplementary Materials File S2 - Exposure Tracking Form.pdf]

Walking Football Injury Surveillance Study (Exposure Card)

Details

Team name: Click or tap here to enter text.

Date range of information being entered (DD/MM/YY to DD/MM/YY): Click or tap here to enter text.

Competitive Matches

1. Number of competitive matches vs. other teams: Click or tap here to enter text.

2. Average competitive match length (minutes): Click or tap here to enter text.

3. Gender category most competitive matches (vs. other teams) were played in:

☐Male ☐Female ☐Mixed Other:Click or tap here to enter text.

4. Age category most competitive matches (vs. other teams) were played in:

☐40+ ☐50+ ☐60+ ☐70+ Other: Click or tap here to enter text.

5. Typical team size (starting the match):

☐5-a-side ☐6-a-side ☐7-a-side Other: Click or tap here to enter text.

Friendly Matches

1. Number of friendly matches vs. other teams: Click or tap here to enter text.

2. Average friendly match length (minutes): Click or tap here to enter text.

3. Gender category most friendly matches (vs. other teams) were played in:

☐Male ☐Female ☐Mixed Other:Click or tap here to enter text.

4. Age category most friendly matches (vs. other teams) were played in:

☐40+ ☐50+ ☐60+ ☐70+ Other: Click or tap here to enter text.

5. Typical team size (starting the match):

☐5-a-side ☐6-a-side ☐7-a-side Other: Click or tap here to enter text.

Training Sessions

1. Number of training sessions: Click or tap here to enter text.

2. Average number of players per training session: Click or tap here to enter text.

3. Average training session length (minutes): Click or tap here to enter text.

4. Most common gender in your training sessions:

☐Male ☐Female ☐Mixed Other:Click or tap here to enter text.

5. Most common age range in your training sessions:

☐40+ ☐50+ ☐60+ ☐70+ Other: Click or tap here to enter text.
