# Supplementary material for: Injury Incidence in Community-Based Walking Football: A Four-Month Cohort Study of 6000+ Hours of Play
Source: Sports (Basel). 2025 May 19;13(5):150. doi: 10.3390/sports13050150 (PMC12115786; doi:10.3390/sports13050150)
Supplement: Supplementary file 1 [file sports-13-00150-s001.zip › Supplementary Materials File S3 - Injury Incidence Calculation Code.pdf]

```r
set.seed(123)

library(tidyverse)

Overall <- data.frame(
  Event = c("Overall", "Training", "Match"),
  Reported.Injs = c(45, 32, 13),
  Reported.HRS = c(6364.55, 6019, 345.55)
)

TL <- data.frame(
  Event = c("Overall", "Training", "Match"),
  Reported.Injs = c(15, 12, 3),
  Reported.HRS = c(6364.55, 6019, 345.55)
)

Med_att <- data.frame(
  Event = c("Overall", "Training", "Match"),
  Reported.Injs = c(30, 20, 10),
  Reported.HRS = c(6364.55, 6019, 345.55)
)


theta <- 5  # Moderate overdispersion assumed here


bootstrap_nb_rate <- function(injuries, exposure, theta, R = 10000) {
  sim_injuries <- rnbinom(R, size = theta, mu = injuries)
  sim_rates <- (sim_injuries / exposure) * 1000
  quantile(sim_rates, c(0.025, 0.975))
}

calculate_bootstrap_rates <- function(df, theta = 5, R = 10000) {
  rate_estimates <- mapply(function(inj, hrs) {
    bootstrap_nb_rate(inj, hrs, theta, R)
  }, inj = df$Reported.Injs, hrs = df$Reported.HRS)

  rate_estimates <- t(rate_estimates)
  colnames(rate_estimates) <- c("Rate.LCL_NB", "Rate.UCL_NB")

  df <- df %>%
    mutate(
      Rate.per.1000.hrs = (Reported.Injs / Reported.HRS) * 1000
    ) %>%
    bind_cols(as.data.frame(rate_estimates)) %>%
    mutate(IR.label.NB = paste0(round(Rate.per.1000.hrs, 2), " (",
                                round(Rate.LCL_NB, 2), "-", round(Rate.UCL_NB, 2),
")"))
  return(df)
}

Overall_boot <- calculate_bootstrap_rates(Overall, theta)
TL_boot <- calculate_bootstrap_rates(TL, theta)
Med_att_boot <- calculate_bootstrap_rates(Med_att, theta)


Overall_boot <- Overall_boot %>%
  select(c(Event, Reported.Injs, Reported.HRS,  IR.label.NB))
print("Overall Injury Rates:")
print(Overall_boot)

TL_boot <- TL_boot %>%
  select(c(Event, Reported.Injs, Reported.HRS,  IR.label.NB))
print("Time Loss Injury Rates:")
print(TL_boot)

Med_att_boot <- Med_att_boot %>%
  select(c(Event, Reported.Injs, Reported.HRS,  IR.label.NB))
```

```
print("Medical Attention Injury Rates:")
print(Med_att_boot)
```
