# Supplementary material for: Injury Incidence in Community-Based Walking Football: A Four-Month Cohort Study of 6000+ Hours of Play
Source: Sports (Basel). 2025 May 19;13(5):150. doi: 10.3390/sports13050150 (PMC12115786; doi:10.3390/sports13050150)
Supplement: Supplementary file 1 [file sports-13-00150-s001.zip › Supplementary Materials File S4 - Injury Rate Ratio Calculation Code.pdf]

```
set.seed(123)

library(tidyverse)

Overall <- data.frame(
  Setting = c("Match", "Training"),
  Injuries = c(13, 32),
  Exposure = c(345.55, 6019)
)

Medical_attention <- data.frame(
  Setting = c("Match", "Training"),
  Injuries = c(10, 20),
  Exposure = c(345.55, 6019)
)

TL <- data.frame(
  Setting = c("Match", "Training"),
  Injuries = c(3, 12),
  Exposure = c(345.55, 6019)
)

# IRR function
bootstrap_nb_irr <- function(inj1, exp1, inj2, exp2, theta = 5, R = 10000) {
  sim1 <- rnbinom(R, size = theta, mu = inj1)
  sim2 <- rnbinom(R, size = theta, mu = inj2)

  rate1 <- sim1 / exp1
  rate2 <- sim2 / exp2

  irr_sim <- ifelse(rate2 == 0, NA, rate1 / rate2)
  irr_sim <- irr_sim[!is.na(irr_sim) & is.finite(irr_sim)]

  point_estimate <- (inj1 / exp1) / (inj2 / exp2)
  ci <- quantile(irr_sim, probs = c(0.025, 0.975))

  return(list(IRR = point_estimate, LCL = ci[1], UCL = ci[2]))
}


data_list <- list(
  Overall = Overall,
  Medical_Attention = Medical_attention,
  Time_Loss = TL
)

# Function looped through data
irr_results <- lapply(names(data_list), function(name) {
  df <- data_list[[name]]
  irr <- bootstrap_nb_irr(
    inj1 = df$Injuries[df$Setting == "Match"],
    exp1 = df$Exposure[df$Setting == "Match"],
    inj2 = df$Injuries[df$Setting == "Training"],
    exp2 = df$Exposure[df$Setting == "Training"],
    theta = 5
  )
  data.frame(
    Type = name,
    IRR = irr$IRR,
    IRR_LCL = irr$LCL,
    IRR_UCL = irr$UCL,
    IRR_Label = paste0(round(irr$IRR, 2), " (", round(irr$LCL, 2), "-", round(irr$UCL,
2), ")")
  )
})

# Combining results
irr_summary <- do.call(rbind, irr_results)
rownames(irr_summary) <- NULL  # Clear inherited row names
```

```
print(irr_summary)
```
